# Pan-RAF and MEK vertical inhibition enhances therapeutic response in non-V600 BRAF mutant cells

**DOI:** 10.1186/s12885-018-4455-x

**Published:** 2018-05-08

**Authors:** Eszter Molnár, Dominika Rittler, Marcell Baranyi, Michael Grusch, Walter Berger, Balázs Döme, József Tóvári, Clemens Aigner, József Tímár, Tamás Garay, Balázs Hegedűs

**Affiliations:** 1 2nd Department of Pathology, Semmelweis University, Budapest, 1091 Hungary; 2 Institute of Cancer Research, Medical University of Vienna, 1090 Vienna, Austria; 3 Department of Thoracic Surgery, Medical University of Vienna, 1090 Vienna, Austria; 4 National Korányi Institute of TB and Pulmonology, Budapest, 1085 Hungary; 5 Department of Thoracic Surgery, Semmelweis University–National Institute of Oncology, Budapest, 1122 Hungary; 6 Department of Experimental Pharmacology, National Institute of Oncology, Budapest, 1122 Hungary; 7 Department of Thoracic Surgery, Ruhrlandklinik, University Duisburg-Essen, 45239 Essen, Germany; 8 HAS-SE Molecular Oncology Research Group, Hungarian Academy of Sciences, Budapest, 1051 Hungary; 9 HAS Postdoctoral Fellowship Program Hungarian Academy of Sciences, Budapest, 1051 Hungary

**Keywords:** Non-V600 BRAF mutation, Sorafenib, AZ628, Selumetinib, Apoptosis, Proliferation, Migration, MEK, RAF, Inhibition

## Abstract

**Background:**

Currently, there are no available targeted therapy options for non-V600 BRAF mutated tumors. The aim of this study was to investigate the effects of RAF and MEK concurrent inhibition on tumor growth, migration, signaling and apoptosis induction in preclinical models of non-V600 BRAF mutant tumor cell lines.

**Methods:**

Six BRAF mutated human tumor cell lines CRL5885 (G466 V), WM3629 (D594G), WM3670 (G469E), MDAMB231 (G464 V), CRL5922 (L597 V) and A375 (V600E as control) were investigated. Pan-RAF inhibitor (sorafenib or AZ628) and MEK inhibitor (selumetinib) or their combination were used in in vitro viability, video microscopy, immunoblot, cell cycle and TUNEL assays. The in vivo effects of the drugs were assessed in an orthotopic NSG mouse breast cancer model.

**Results:**

All cell lines showed a significant growth inhibition with synergism in the sorafenib/AZ628 and selumetinib combination. Combination treatment resulted in higher Erk1/2 inhibition and in increased induction of apoptosis when compared to single agent treatments. However, single selumetinib treatment could cause adverse therapeutic effects, like increased cell migration in certain cells, selumetinib and sorafenib combination treatment lowered migratory capacity in all the cell lines. Importantly, combination resulted in significantly increased tumor growth inhibition in orthotropic xenografts of MDAMB231 cells when compared to sorafenib - but not to selumetinib – treatment.

**Conclusions:**

Our data suggests that combined blocking of RAF and MEK may achieve increased therapeutic response in non-V600 BRAF mutant tumors.

**Electronic supplementary material:**

The online version of this article (10.1186/s12885-018-4455-x) contains supplementary material, which is available to authorized users.

## Background

The RAS/RAF/MEK/ERK pathway consists of numerous signaling elements that, when affected by mutations, can promote tumorigenesis and tumor progression [1]. One of the most frequent cause of RAS/RAF/MEK/ERK pathway aberrant activation is BRAF gain-of function mutation (7–15%) [2, 3]. More than 80% of BRAF mutations are single amino acid substitutions of valin for glutamic acid in position 600 (V600E). Additionally, BRAF V600 K mutations also occur frequently in BRAF-mutated melanoma (7–19%) [3]. However incidence of non-V600 BRAF mutation is not negligible in certain cancer types. Lung adenocarcinoma, melanoma and colorectal cancer with BRAF mutation showed 50–86, 34 and 23% non-V600 BRAF mutation frequency, respectively [4–6]. Despite the notable number of the cases, there is no effective targeted therapy against non-V600 BRAF mutant tumors.

RAS/RAF/MEK/ERK pathway vertical inhibition in treatment of V600 BRAF mutant melanoma is already approved in clinical practice. Compared to V600 mutant BRAF single inhibition, combination inhibition of V600 mutant BRAF and MEK displayed elevated progression free and overall survival in clinical trials with metastatic BRAFV600 mutant melanoma patients [7, 8]. Also recent studies have shown that in NRAS mutant melanoma, RAS/RAF/MEK/ERK pathway vertical inhibition leads to a synergistic inhibitory effect [9, 10]. Pan-RAF and MEK double treatment proved to be an effective therapeutic strategy in vitro in NRAS mutant melanomas, when RAS/RAF/MEK/ERK pathway is activated and dependence of proliferation and survival on this pathway is demonstrable [9]. Furthermore MEK and ERK1/2 combination inhibition induced a high level of apoptosis in NRAS mutant melanoma cell lines [10]. Also in KRAS mutant colorectal and lung cancer cell lines RAF and MEK combination inhibition abrogates ERK1/2 signaling and triggers apoptosis [11]. Enhanced therapeutic effects of MEK inhibitor and pan-RAF inhibitor (sorafenib) combination therapies have already been described in hepatocellular, thyroid, lymphoma and renal carcinoma [12–15].

Non-V600 BRAF mutant tumors often carry concomitant mutations in RAS or PI3K [6]. NRAS or KRAS mutations frequently occur concurrently with kinase dead BRAF mutations and play a role in MAPK pathway activity maintenance via CRAF [6, 16, 17]. Also the MAPK pathway could be still activated through dimerization of BRAF with reduced kinase activity and wild-type CRAF [2, 6, 18]. Since in non-V600 BRAF mutant tumor cells the MAPK pathway could still play a dominant role via CRAF in proliferation, survival and migration, we hypothesized that vertical inhibition of RAF and MEK may lead to enhanced therapeutic effects as found previously in V600 BRAF mutant cells.

The aim of our study was to investigate the effect of pan-RAF inhibitors (sorafenib and AZ628) and MEK inhibitor (selumetinib) combination treatment on non-V600 BRAF mutant tumor cell lines with various BRAF activities (Table 1). Sorafenib has broad preclinical activity across tumor types. While sorafenib had the lowest IC50 values against CRAF and wild-type BRAF, 6 and 22 nM, respectively, and thus can be considered a pan-RAF inhibitor, it also targets a variety of other kinases including VEGFR-1, VEGFR-2, VEGFR-3, PDGFR, c-Kit, Flt-3 and RET. [19, 20]. AZ628 has strong selectivity for RAF kinases with an IC50 value of 30 nM for BRAF V600E and wild-type CRAF and of 100 nM for wild-type BRAF [21]. Selumetinib is a non-ATP competitive and highly selective MEK 1/2 inhibitor. In clinical trials with selumetinib, patients harboring RAS/RAF mutations had higher objective response rate than patients with wild-type RAS/RAF [22].

**Table 1:** Cell lines by tissue type, BRAF and RAS mutational status

Cell lines	Tissue	BRAF	BRAF activity	RAS (N/K)	Reference
A375	melanoma	V600E	high	wild type	[45]
CRL5885	lung	G466 V	low	wild type	[25]
WM3629	melanoma	D594G	low	G12D (N)	[27]
WM3670	melanoma	G469E	low	G12D (N)	[27]
MDAMB231	breast	G464 V	intermediate	G13D (K)	[46]
CRL5922	lung	L597 V	intermediate	Q61K (N)	[47]

Here, we report that in preclinical models of non-V600 BRAF mutant tumors, combination of sorafenib/AZ628 and selumetinib shows enhanced therapeutic effects compared to single treatment.

## Methods

### Reagents and cell lines

Selumetinib, sorafenib and AZ628 were obtained from Selleck Chemicals (Houston, TX), LC Laboratories (Woburn, MA) and Sigma-Aldrich (St. Louis, MO), respectively. Six human tumor cell lines were used in the experiments. Cell lines A375 (ATCC, CRL-1619), CRL5885 (ATCC, CRL-5885), CRL5922 (ATCC, CRL-5922) and MDAMB231 (ATCC, HTB-26) are available from ATCC. WM3629 (Coriell Cat# WC00117, RRID:CVCL_C275), WM3670 (Coriell Cat# WC00119, RRID:CVCL_6799) cell lines were obtained from the Wistar Institute **(**Table 1). Activation status of the BRAF mutations are from Zheng et al., 2015 [6]. Cell lines have no known additional mutations in PI3K or PTEN [23–27].

All cell lines were cultured in DMEM (Lonza, Switzerland; with 4500 mg/dm^3^ glucose, piruvate and L-glutamine) supplemented with 10% foetal calf serum (Gibco) and 1% penicillin-streptomycin-amphotericin (Lonza). Cells were maintained in tissue culture flasks at 37 C^o^ with 5% CO_2_.

### Sulforhodamine B (SRB) assay and drug combinatory index assessment

Long-term antiproliferative effects of sorafenib and selumetinib treatment were assessed by performing clonogenic SRB (Sigma-Aldrich, St. Louis, MO) assays. Briefly, 2000 cells (WM3629, WM3670, CRL5885, CRL5922) or 250 cells (A375, MDAMB231) were plated in 24-well plate format and cultured overnight. Next day, the cells were treated with different concentrations of sorafenib or AZ628 and selumetinib and with combined treatment for 10 days. After 10 days, cell monolayers were fixed with 10% trichloroacetic acid and stained for 15 min with SRB. Excess stain was discarded and cells were washed with 1% acetic acid solution. Stained cells were dissolved in 10 mM Tris-HCl pH 8 and OD was measured at 570 nm using a microplate reader (EL800, BioTec Instruments, Winooski, VT). Interactions between drugs were tested on the basis of calculating the combination index (CI) according to Chou and Talalay [28] with CompuSyn software (ComboSyn Inc). CI values CI < 1, CI = 1 or CI > 1 represents synergism, additive effects, and antagonism, respectively.

### Immunoblot analysis

Immunoblot analysis was performed to demonstrate the effect of selumetinib and sorafenib treatment on the activation of CRAF, Erk1/2, Akt (at Ser473) and S6 protein. Induction of apoptosis upon selumetinib and sorafenib/AZ628 treatment was detected by total PARP and cleaved PARP. Cells were seeded in six-well plates and maintained overnight. Next day selumetinib and sorafenib/AZ628 or combination treatment were applied for 4 h or 48 h to determine the activation changes of CRAF, Erk, Akt and S6 or cleavage of PARP, respectively. For p-CRAF/CRAF, p-Erk1/2/Erk1/2, p-Akt/Akt, p-S6/S6 detection ice-cold 6% trichloroacetic acid (TCA) were used to precipitate cells. Then cells were centrifuged for 15 min at 12000×*g* at 4 °C. Modified Läemmli-type sample buffer containing 90 mM Tris-HCl, pH 7.9, 2% SDS, 10% glycerol, 5 mM EDTA, 125 mg/ml urea, 100 mM dithiothreitol (DTT), 0.02% bromophenol blue was used to dissolve protein pellets. Protein concentrations were measured by the modified Lowry method using bovine serum albumin as standard. To detect total/cleaved PARP cells were lysed with RIPA Buffer (Thermo Scientific, Waltham, MA) supplemented with 1% Halt Protease Inhibitor Single-Use Cocktail (Thermo Scientific). Total protein concentrations were measured with Pierce BCA Protein Assay kit (Thermo Scientific). Protein samples were separated by SDS-PAGE (10%) and transferred to PVDF membranes (Thermo Scientific). Primary antibodies to antiPARP/cleaved-PARP (Merck Millipore AM30, Cell Signaling; #9541) and anti p-Erk1/2/Erk1/2, p-Akt/Akt, p-S6/S6, p-CRAF/CRAF (Cell Signaling; #9101, #9102, #4058, #9272 #2215, #2217, #9427, #9422, respectively) and as loading control anti β-tubulin or β-actin (Cell Signaling #2128 and #4970), overnight at 4 °C in a dilution of 1:1000 were applied. Secondary HRP-conjugated anti-rabbit or anti-mouse antibody (Jackson ImmunoResearch, West Grove, PA) was used (1:10000, 1 h) at room temperature. Pierce ECL Western Blotting Substrate (Thermo Scientific) was used to visualize the protein bands.

### TUNEL assay

Cells were seeded in 24 well plates (50,000 cells/well) and next day selumetinib or sorafenib or a combined treatment were applied. After 48 h of treatment 4% buffered formalin was used to fix the cells. Labelling of terminal deoxynucleotidyl transferase—mediated dUTP nick end (TUNEL) was performed according to the supplier’s recommendation (Roche Diagnostics, Basel, Switzerland). DAPI stained and TUNEL positive nuclei on at least three 10× microscopic fields were counted to quantify the images.

### Cell cycle analysis

To determine cell cycle change upon selumetinib and sorafenib treatment, cells were treated with the inhibitors for 48 h in 6-well plates. Cell cycle analysis was carried out as described earlier [29]. Briefly, cells were trypsinized and lysed before staining with DAPI for 5 min at 37 °C. After adding the stabilization buffer, samples was loaded onto an 8-well NC slide. NucleoCounter NC-3000™ system (Chemometec, Allerod, Denmark) was used to quantify cellular fluorescence.

### Time-lapse video microscopy

Video microscopy measurements were performed and analyzed as described previously [30]. The parameter migrated distance is calculated by averaging for each cell the displacement for the 48–60 h interval after treatment, in at least three independent experiments and three microscopic fields.

### Mammary xenografts of MDAMB231 breast cancer cells

Animal experiments were carried out at the Department of Experimental Pharmacology, National Institute of Oncology, Budapest, Hungary and the animal-model experiments were conducted following the standards and procedures approved by the Animal Care and Use Committee of the National Institute of Oncology, Budapest (license number: PEI/001/2574–6/2015). 14-weeks-old female NSG mice were used as animal model, since previous work described NSG mice as a suitable model for study human breast cancer [31]. Mice were bred and maintained in specific pathogen-free facility. MDAMB231 cells (2 × 10^6^ in 50 μl serum-free DMEM) were injected into the mammary fat pad of female NSG mice. Two weeks after injection, mice were randomly and evenly divided into four groups (10 mice/group) and treated with vehicle, 25 mg/kg sorafenib and 10 mg/kg selumetinib or both intraperitoneally (i.p) every day except weekends for 18 days. Controls received equivalent amounts of DMSO as treated animals. All animals were included in the analysis. The changes of the body weight were also determined throughout the study (Fig. 5b). No adverse events were observed during the experiment. Tumors were measured with caliper twice a week and tumor sizes were calculated with the formula for the volume of a prolate ellipsoid (length x width^2^ x (4π/3)) and then transformed into relative values (V) using the formula: V = Vt/V0, where V0 is the initial tumor volume and Vt is the tumor volume at the indicated time point. Eighteen days after the first treatment, mice were euthanized and the tumor tissue was removed and weighed.

### Statistics

All statistical analyses were performed in GraphPad Prism 5 (GraphPad Software Inc., USA, San Diego, CA). One-way ANOVA followed by Tukey’s post hoc test was used to establish whether significant differences existed between groups. Differences were considered significant at *p* < 0.05.

## Results

### Synergistic effect of sorafenib and selumetinib combination in non-V600 mutant cell lines

**Fig. 1:**
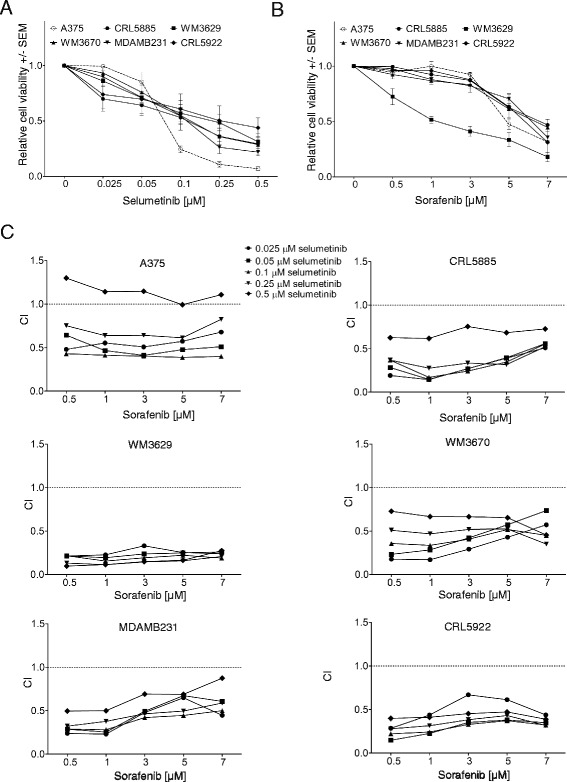
Growth inhibition assay with single agent or combination of sorafenib and selumetinib. **a** Cell lines were treated with different concentrations of selumetinib for ten days (mean values +/− SEM). **b** Cell lines were treated with different concentrations of sorafenib for ten days (mean values +/− SEM). **c** Combination indices (CI) were calculated by CompuSyn Software from the data of viability assays of selumetinib and sorafenib combination treatment (Additional file 1: Figure S1). CI values less than 1 indicate synergy while values equal to or more than 1 represent additive or antagonistic effect, respectively

Growth inhibition assays were performed with single agents or a combination of sorafenib and selumetinib on a panel of non-V600 mutant human cell lines and V600E BRAF mutant A375 cell lines (Table 1). Among these cell lines, the V600E BRAF mutant A375 cell line was the most sensitive to selumetinib treatment, while non-V600 BRAF mutant cells showed similar sensitivity to single selumetinib treatment (Fig. 1a). The growth inhibitory effect of sorafenib was also similar among the cell lines, except the double mutant WM3629, which was more sensitive compared to the other cells (Fig. 1b). To identify the synergistic effect of sorafenib and selumetinib, dose–response curves were established by viability assays following 10 days selumetinib and sorafenib combination treatment (Additional file 1: Figure S1). Combination indices (CI) were calculated by CompuSyn Software from the data of viability assays of combination treatment. All cell lines - including the V600E BRAF mutant A375 at lower selumetinib concentration - showed lower CI value than 1 indicating synergistic interaction in combination treatment (Fig. 1c).

### Effect of sorafenib and selumetinib combination treatment on signaling of CRAF, Erk1/2, Akt and S6 kinases

**Fig. 2:**
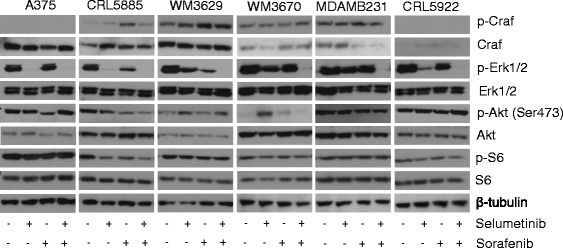
Signaling in sorafenib and/or selumetinib treated cells. Cells were treated with 1 μM of sorafenib and 50 nM of selumetinib as indicated for 4 h. Phosphorylation inhibition of Erk1/2 is enhanced by combination treatment. Single selumetinib treatment could increase Akt activation in WM3629 and WM3670 cell lines. Total expression of CRAF could be induced in non-V600 BRAF mutant cells but not in V600E BRAF mutant A375 cell. β-tubulin served as loading control. Blots are representative images from three independent experiments

To investigate the effect of selumetinib and sorafenib or combined treatment on CRAF, Erk1/2, Akt and S6 activation, cell lines were treated with single agents or the combination and were analyzed by Western blotting (Fig. 2). We observed a decrease of Erk1/2 activation in all non-V600 BRAF mutant cell lines after both sorafenib and selumetinib treatment. In comparison with the single agent treatment, combination of drugs caused even further decreases in p-Erk1/2 levels in all cell lines, except A375, where selumetinib treatment caused complete Erk1/2 deactivation. Our data suggest that combination treatment could be more effective for the blocking of the MAPK pathway in non-V600 BRAF mutant cell lines. In order to measure the effect of sorafenib and selumetinib on Akt/mTOR pathway, we evaluated the level of total and phosphorylated Akt and S6 in the cells. Effect of combination treatment on Akt and S6 activation was not uniform in all cell lines. We observed slightly increased p-Akt levels in WM3629 and CRL5922, however in the other cell lines Akt activation decreased or not altered. Also combination of drugs decreased or not changed the activation of S6 among the cell lines. Notably, in WM3629 and WM3670 melanoma cells single selumetinib treatment also caused increased activation of Akt. Furthermore, in non-V600 BRAF mutant tumors combination treatment could induce total CRAF expression but not in V600E BRAF mutant A375. Also we observed weak induction of CRAF activation upon sorafenib or selumetinib at Ser338 in CRL5885, WM3629, WM3670 and MDAMB231.

### Sorafenib and selumetinib combination treatment leads to apoptosis and G0/G1 cell-cycle arrest in non-V600 mutant cell lines

**Fig. 3:**
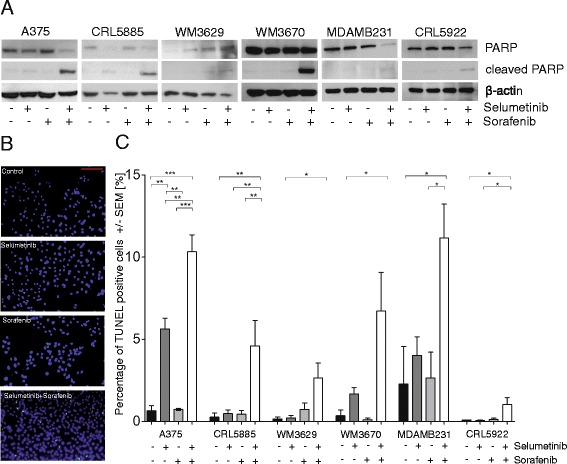
Apoptosis induction by sorafenib and/or selumetinib treatment. Higher apoptotic response of non-V600 BRAF mutant cell lines to the combination than to single agent treatment determined by **a** cleaved PARP/PARP and **b**, **c** TUNEL assay. **b** Representative pictures of the WM3670 cell line with TUNEL (green) and DAPI (blue) staining. Scale bar = 100 μm. **c** Proportion of TUNEL positive cells upon sorafenib and/or selumetinib treatment. Cells were treated with sorafenib (3 μM), selumetinib (100 nM) or the combination for 48 h (*p ≤ 0.05, * * *p* ≤ 0.01, * * * *p* ≤ 0.001)

PolyADP-ribose polymerase cleavage (PARP/cleaved PARP) immunoblot assay (Fig. 3a) and TUNEL staining (Fig. 3b and c) was used to determine whether growth inhibition was due to induction of apoptosis in our panel of BRAF mutated cell lines. Treatment with selumetinib or sorafenib alone had either no (WM3670, MDAMB231) or minimal (A375, CRL5885, CRL5922) effects on cleavage of PARP, whereas combined treatment resulted in a pronounced increase of PARP degradation (Fig. 3a). The TUNEL assay data further confirmed this finding. We observed that combination treatment significantly increase the level of apoptotic cells compared to single treatment. Interestingly, also A375 cell line showed an elevated apoptotic effect in response to combined treatment. These results indicate that RAF and MEK1/2 vertical inhibition increases the potential to trigger apoptosis in non-V600 and also V600E BRAF mutant cells. Also cell cycle analyses revealed that combination treatment reduced cell proliferation by arresting cell cycle in G0/G1. Furthermore, we observed elevated subG1 cell population upon combination treatment in CRL5885 and MDAMB231 cells (Additional file 2: Figure S2).

### Migration is inhibited by sorafenib and selumetinib combination treatment in non-V600 mutant cells

**Fig. 4:**
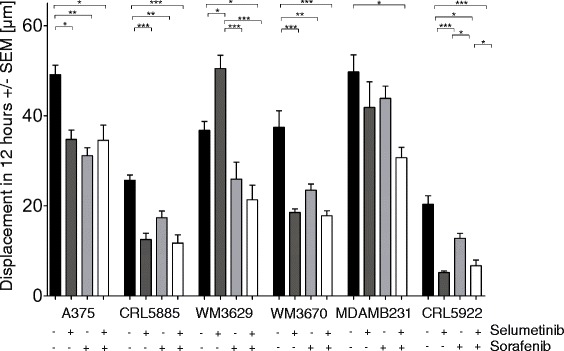
Cell migration analysis by long-term video microscopy. Sorafenib (3 μM) and selumetinib (500 nM) combination treatment decrease migratory ability in non-V600 BRAF mutant cell lines. Average net displacement was determined between 48 and 60 h after the treatment. Data is shown as mean +/− SEM of at least three independent measurements (*p ≤ 0.05, * * *p* ≤ 0.01, * * * *p* ≤ 0.001)

Migratory potential of the cells was evaluated via video microscopy measurements [30]. Single treatment of selumetinib decreased the migratory ability of most non-V600 BRAF mutant cell lines. Interestingly, in WM3629 melanoma cells selumetinib increased migration. Sorafenib also decreased migration among cell lines. Combination treatment has no significantly stronger effect on migration than single agent treatment in neither of cell lines, however in WM3629 and MDAMB231 we observed statistically significant decrease in migration only in combination treatment group when compared to control group (Fig. 4).

### Combination of selumetinib and sorafenib therapy enhances tumor growth inhibition in MDAMB231 xenograft compared to single agents with negligible toxicity

**Fig. 5:**
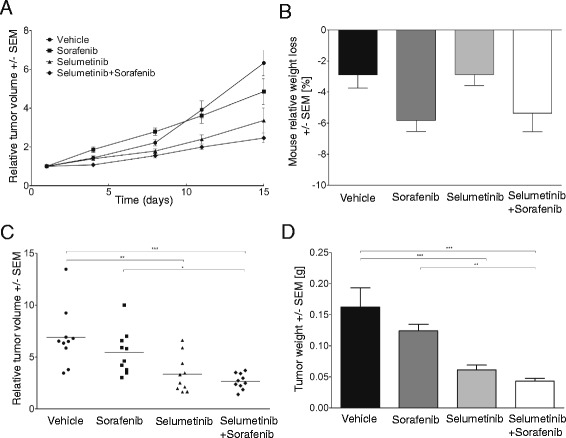
In vivo tumor growth in MDAMB231 xenografts upon sorafenib and/or selumetinib therapy. **a** Tumor volume was determined twice/weekly using caliper. **b** Mouse weight loss (%) at day 18, compared to the beginning of the treatment. **c** Relative tumor volume of MDAMB231 mammary xenografts at day 18. **d** Tumor weights of MDAMB231 mammary xenografts at day 18. Each group consisted of 10 animals. Data is shown as the mean +/− SEM (*p ≤ 0.05, * * *p* ≤ 0.01, * * * *p* ≤ 0.001)

We examined the effect of selumetinib, sorafenib or combined treatment on in vivo growth of MDAMB231 cells transplanted into 14-weeks-old NSG mice. MDAMB231 cells were injected into mammary fat pads of female mice and animals were monitored for the growth of palpable tumor at the site of injection. Two weeks after cell injection, animals received either vehicle as control or selumetinib at 10 mg or sorafenib at 25 mg or both (per kg body weight) daily, except weekends by i.p. injection. We observed that both sorafenib or selumetinib treatments reduced tumor growth, however the combination resulted in an enhanced growth inhibitory effect. Tumor growth inhibition was significantly greater in the combination group compared to vehicle and sorafenib (Fig. 5a,c and d). To assess the toxicity associated with the drug treatment, body weights were monitored throughout the course of the study. Body weights at day 18 were not significantly different from day 1 body weight in each group of animals. Also body weight losses were not significantly different between treatment groups (Fig. 5b).

### Combination of the selective pan-Raf inhibitor AZ628 and selumetinib also increases growth inhibition and PARP cleavage activity compared to single treatment in non-V600 BRAF mutant cells

**Fig. 6:**
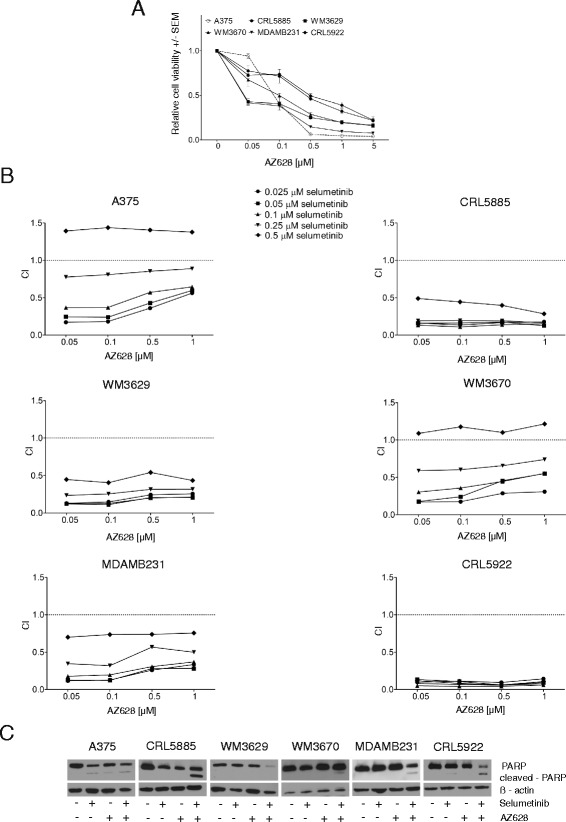
Growth inhibition and PARP levels upon combination treatment of selumetinib and AZ628. **a** Cell lines were treated with different concentrations of AZ628 for 10 days (mean values +/− SEM). **b** Combination indices (CI) were calculated by CompuSyn Software from the data of viability assays of selumetinib and AZ628 combination treatment (data not shown). **c** PARP cleavage activity upon selumetinib and AZ628 single agent or combination treatment

Since sorafenib also has inhibitory effect on several other kinases, we performed growth inhibition and PARP cleavage assay with a highly selective pan-RAF inhibitor (AZ628) on our panel of non-V600 BRAF mutant cells. We found that WM3629 and MDAMB231 showed the highest sensitivity to AZ628 single treatment (Fig. 6a). Also combination treatment revealed significant synergism between selumetinib (below 0.5 μM concentration) and AZ628 in all cell models tested (Fig. 6b). Furthermore, the combination of selumetinib with AZ628 enhanced cleavage of PARP in CRL5885, WM3670, MDAMB231 and CRL5922 and also decreased the total PARP level in WM3629 cell line (Fig. 6c).

## Discussion

Currently there are no approved targeted therapeutic options for non-V600 BRAF mutant tumors. In our study we have demonstrated that in non-V600 BRAF mutant cells the combination of sorafenib and selumetinib treatment synergistically enhances growth inhibition compared to single treatment. Combination treatment also improves apoptosis induction and Erk1/2 inhibition compared to monotherapy and, moreover, also decreases migratory capacity of the cells. We have also performed growth inhibition and PARP cleavage assay with highly selective pan-RAF inhibitor AZ628 to confirm that effects of sorafenib treatment on non-V600 BRAF mutant cells are due to its pan-RAF inhibitor activity. We found that also AZ628 and selumetinib combination treatment resulted in synergistically increased growth inhibition and PARP cleavage in non-V600E BRAF mutant cells.

While our study is the first report on sorafenib/AZ628 and selumetinib combination, there are already studies on non-V600 BRAF mutant lung cancer cell lines using the BRAF V600E mutant selective inhibitors dabrafenib or vemurafenib and the MEK inhibitor trametinib [32, 33]. In the H1666 (CRL5885) and H1395 non-V600 BRAF mutant lung cancer cell lines and the H508 colorectal cancer cell line dabrafenib and trametinib combination treatment showed enhanced anti-proliferative effects and caspase3/7 activation, however these results were not significant in all cell lines [33]. In the H1755 non-V600 BRAF mutant lung cancer cell line vemurafenib and trametinib combination treatment caused a small but significant increase in apoptosis when compared to either single agent [32]. It was also shown that in low but not in high activity BRAF mutant melanoma cell lines (WM3670, WM3629) sorafenib treatment could reduce tumor growth and induce apoptosis [34]. We observed similar effects upon sorafenib treatment in these cells; however, adding selumetinib further increased inhibition of tumor growth and migration as well as enhanced the apoptosis inducing effects of sorafenib.

Targeted therapy could induce adverse effects on parallel signaling pathways. It was previously described that sorafenib treatment tends to elevate Akt activation in cell lines with KRAS or BRAF mutations [35]. Also MEK inhibition could increase p-Akt level in RAF mutant cells [36] and KRAS mutant cells [37]. We observed that after 4 h treatment of selumetinib p-Akt level increased in WM3670 and WM3629 cells that have low activity non-V600 BRAF and additional NRAS mutation. Furthermore, we found that in the WM3629 cell line selumetinib caused significantly elevated migration which could be diminished by adding sorafenib. Our findings suggest that the potentially adverse signaling effects of selumetinib could be reversed by adding sorafenib.

We found significant tumor growth inhibition in the in vivo xenograft model of the MDAMB231 cell line upon sorafenib and/or selumetinib treatment. Although the enhanced tumor growth inhibition did not reach statistical significance between the selumetinib and the combination treatment group, in the combination group the variance between relative tumor volumes was significantly lower than in the selumetinib group (*p* = 0.017, F-test). This observation indicates that combination treatment reduced the number of weak-responders in comparison to selumetinib single treatment. Furthermore, a recent study showed that the selumetinib and sorafenib combination treatment almost eliminated tumor growth and reduced metastatic pulmonary tumor burden in in vivo experiments with MDAMB231 cells [38]. Nevertheless, additional in vivo preclinical models need to be investigated to clarify whether there is a therapeutic benefit from the combination in non-V600 BRAF mutant tumors.

There is an urgent and unmet need to find effective therapeutic treatment for non-V600 BRAF mutant tumors. Recent studies show that advanced melanoma and colorectal cancer patients with non-V600 BRAF mutation have longer overall survival compared those with both V600E BRAF mutant and wild-type BRAF [39, 40]. However, in non-V600 BRAF mutant melanoma patients the BRAF V600E selective inhibitors (vemurafenib or dabrafenib) had only disease progression as the best response to therapy [39]. Importantly, advanced lung cancer patients with non-V600 BRAF mutation has worse overall survival than V600E mutant patients [5, 41, 42]. Of note, it was reported in a case study, that a female patient with non-V600 BRAF (G469R) mutated lung adenocarcinoma showed dramatic response to sorafenib treatment [43].

Based on our preclinical findings, sorafenib treatment combined with selumetinib might result in enhanced therapeutic effect also in patients with non-V600 BRAF mutant tumors. Since our study investigated only one in vivo preclinical model, further studies are warranted to confirm whether combinatorial treatment delivers additional benefit compared to monotherapy. Regarding the safety of the proposed combination, a recent phase I study with sorafenib and selumetinib combination showed promising effectiveness and tolerable adverse effects in advanced hepatocellular carcinoma patients [44].

## Conclusion

Currently, there are no approved targeted therapies for non-V600 BRAF mutant cancer patients. Our in vitro data suggests that combination inhibition of RAF and MEK with sorafenib/AZ628 and selumetinib, respectively, should be further explored as a potential approach for inhibiting tumor growth in non-V600 BRAF mutant malignancies.

## Additional files

Additional file 1: **Figure S1.** Dose–response curves for sorafenib without or with the indicated selumetinib concentrations. Viability was measured by SRB assay after 10 days drug exposure and normalized to untreated controls (mean values +/− SEM). The respective combination indices (CI) were calculated by CompuSyn Software and are shown in Fig. 1c. (EPS 4764 kb)

Additional file 2: **Figure S2.** Analysis of cell cycle after treatment with selumetinib or sorafenib and combination. Cells were treated with selumetinib (50 nM), sorafenib (1 μM), alone or in combination for 48 h. C – control, Se – selumetinib, So – sorafenib, Se + So – selumetinib + sorafenib. (EPS 8097 kb)

## Abbreviations

SRB: Sulforhodamine B
TUNEL: Terminal deoxynucleotidyl transferase-mediated deoxyuridine triphosphate-biotin nick end labeling
